# Folic Acid Supplementation Inhibits Proliferative Retinopathy of Prematurity

**DOI:** 10.3390/biom15020309

**Published:** 2025-02-19

**Authors:** Shen Nian, Yan Zeng, Katarina E. Heyden, Gaël Cagnone, Hitomi Yagi, Myriam Boeck, Deokho Lee, Victoria Hirst, Zhanqing Hua, Jeff Lee, Chaomei Wang, Katherine Neilsen, Jean-Sébastien Joyal, Martha S. Field, Zhongjie Fu

**Affiliations:** 1 Department of Ophthalmology, Boston Children’s Hospital, Harvard Medical School, Boston, MA 02115, USA; shennian@xiyi.edu.cn (S.N.); yan.zeng@childrens.harvard.edu (Y.Z.); hitomi.yagi@childrens.harvard.edu (H.Y.); deokho.lee@childrens.harvard.edu (D.L.); victoria.hirst@childrens.harvard.edu (V.H.); anthea.hua@childrens.harvard.edu (Z.H.); chaomei.wang@childrens.harvard.edu (C.W.); katherine.neilsen@childrens.harvard.edu (K.N.); 2 Department of Pathology, Xi’an Medical University, Xi’an 710021, China; 3 Division of Nutritional Sciences, Cornell University, Ithaca, NY 14853, USA; keh67@cornell.edu (K.E.H.); mas246@cornell.edu (M.S.F.); 4 Centre Hospitalier Universitaire Sainte-Justine Research Center, Montreal, QC H3T 1C5, Canadajs.joyal@umontreal.ca (J.-S.J.); 5 Department of Ophthalmology, Keio University School of Medicine, Tokyo 160-8582, Japan; 6 Eye Center, Medical Center, Faculty of Medicine, University of Freiburg, 79106 Freiburg, Germany; 7 Department of Pediatrics, Faculty of Medicine, Université de Montréal, Montreal, QC H3C 3J7, Canada

**Keywords:** retinopathy of prematurity, folate, neovascularization, angiogenesis, oxygen-induced retinopathy, retina

## Abstract

Background: Retinopathy of prematurity (ROP) is the major cause of blindness in children. It is a biphasic disease with retinal vessel growth cessation and loss (Phase I) followed by uncontrolled retinal vessel growth (Phase II). Folate is an essential nutrient for fetal development and growth. Premature infants have a high risk for folate deficiency. However, the contribution of folate to ROP risk remains unknown. Methods: In mouse oxygen-induced retinopathy (OIR), the nursing dams were fed with a folic acid-deficient or control diet after delivery until the end of hyperoxia. Alternatively, pups received direct injection of either folic acid or vehicle during Phase I hyperoxia. Genes involved in the folate cycle and angiogenic responses were examined using real-time PCR. Total retinal folate levels were measured with the *Lactobacillus casei* assay. Results: Maternal folic acid deficiency in early life exacerbated pathological retinal vessel growth, while supplementation with folic acid suppressed it. Genes involved in the folate cycle were downregulated in Phase I OIR retinas and were highly expressed in Müller glia. Folic acid reduced pro-angiogenic signaling in cultured rat retinal Müller glia in vitro. Conclusions: Appropriate supplementation of folic acid might be a new and safe treatment for ROP at an early stage.

## 1. Introduction

Neovascular retinopathy of prematurity (ROP) can cause blindness in premature infants [1]. Current treatments targeting late-phase neovascularization may either damage the developing neurosensory retina (laser photocoagulation) or systemically suppress the developing vasculature in other organs (anti-vascular endothelial growth factor (VEGF) therapies) [2,3,4]. We need to prevent neovascularization with safe interventions, such as the provision of essential nutrients missing after preterm birth, as inadequate nutrition is a significant factor contributing to ROP [5].

Folate (also known as vitamin B9) is an essential micronutrient. Humans are entirely dependent on dietary sources for their supply [6], as folate is critical for fetal development. During pregnancy, low dietary and circulating folate are associated with increased risk of preterm delivery, low birth weight, and fetal growth retardation [6,7]. Folate deficiency occurs in premature infants as the demands for growth exceed the intake of folate. Blood folate level drops rapidly after birth, and low serum folate level persists for up to 6 months of age in premature infants [8,9,10,11]. Preterm infants suffer from folic acid deficiency due to the insufficient amount of folic acid in human breast milk and preterm formula, which falls short of the recommended intake for preterm infants [9,12]. Maternal dietary folic acid supplementation reduces defects in the brain and the spine [13]. Folic acid therapy works well in premature infants with anemia [11], and premature infants (≤800 g) who remain severely anemic are more likely to develop ROP than less anemic infants [14]. However, the association between folic acid and ROP is still unknown and needs to be explored.

Folate deficiency and impaired folate pathways have serious consequences for the visual system and are implicated in many eye diseases [15]. Folate deficiency leads to nutritional amblyopia, nutritional optic neuropathy, cataracts, corneal epitheliopathy, and chronic conjunctivitis [15]. Mutations in methylenetetrahydrofolate reductase (MTHFR, a key enzyme for producing the biologically active form of folate, L-methylfolate) and low blood levels of folate are associated with retinal vein occlusion and glaucoma [16,17,18]. A recent report also showed that a severe folate and vitamin B12 deficiency caused massive preretinal hemorrhage and a sudden decrease in vision in a 22-year-old female [19]. Experimentally, a maternal folic acid-deficient diet in mice causes severe abnormalities in both anterior and posterior segments of the eye, as well as choroidal vessels during gestation [20,21]. We aimed to examine the impact of folate deficiency on retinal neovascularization using the well-established oxygen-induced retinopathy (OIR) [22] mouse model to mimic proliferative ROP. We will evaluate if early folic acid supplementation may prevent ROP.

## 2. Materials and Methods

### 2.1. Study Approval

All animal studies adhered to the Association for Research in Vision and Ophthalmology Statement for the Use of Animals in Ophthalmic and Vision Research and were approved by the Institutional Animal Care and Use Committee at Boston Children’s Hospital (#00001619, approved on 18 February 2022).

### 2.2. Mouse Model of OIR

**Figure 1:**
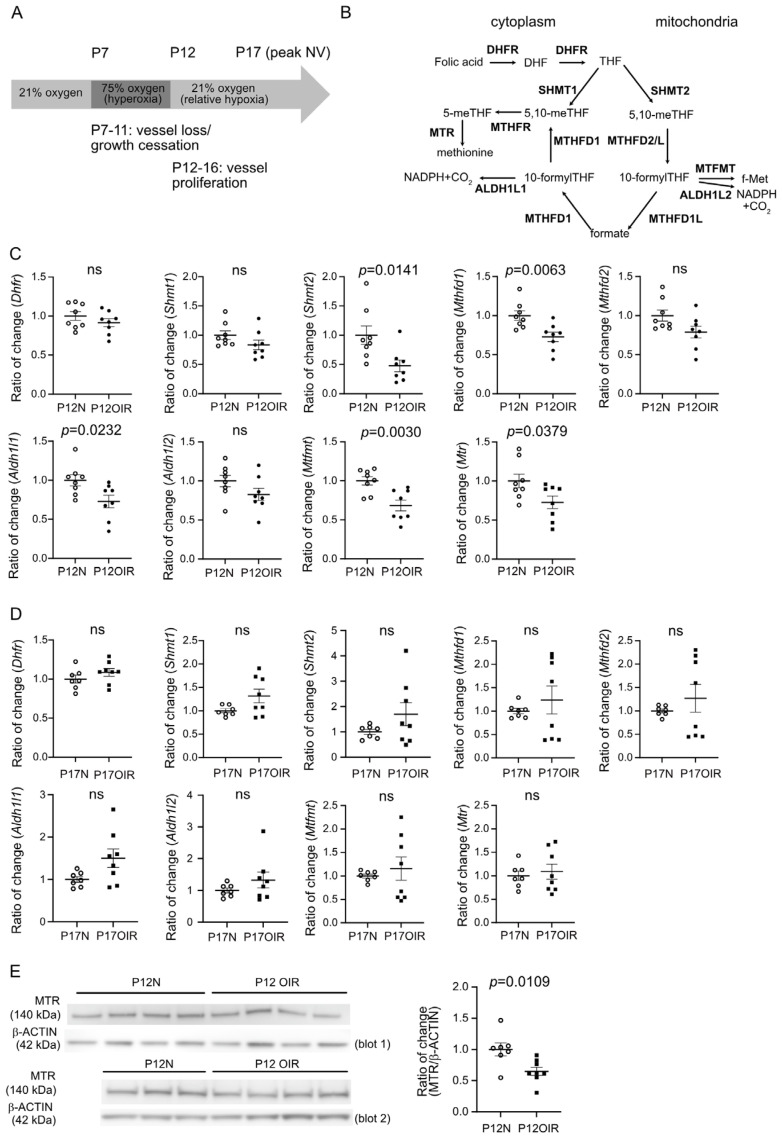
Expression of metabolic genes involved in folate cycle at OIR vs. normal retinas. (**A**) Schematics of mouse model of oxygen-induced retinopathy (OIR). Hyperoxia-induced vessel loss and growth cessation and relative hypoxia-induced uncontrolled vessel proliferation. Peak neovascularization (NV) occurs at P17. (**B**) Schematics of the folate cycle. Metabolic enzymes were highlighted in bold. Dihydrofolate reductase (DHFR), serine hydroxymethyltransferase (SHMT1, SHMT2), methenyltetrahydrofolate cyclohydrolase (MTHFD1, MTHFD2), NAD-dependent methylenetetrahydrofolate dehydrogenase 2-like protein (MTHFD2L), 10-formyltetrahydrofolate dehydrogenase (ALDH1L1), aldehyde dehydrogenase 1 family, member L2 (ALDH1L2), mitochondrial methionyl-tRNA formyltransferase (MTFMT), methionine synthase (MTR), and methylenetetrahydrofolate reductase (MTHFR). (**C**,**D**) qPCR of metabolic genes involved in the folate cycle. Retinas were isolated from P12 (**C**) and P17 (**D**) OIR vs. normal control mice. *n* = 8 mice per group. (**E**) Western blot of MTR in P12 OIR vs. control retinas. *n* = 7–8 mice per group, two independent litters for each group. β-ACTIN was used as internal control. Ratio of change was calculated over P12N (averaged value of the intensity of MTR over β-ACTIN) on the same blot. Analysis was conducted using combined values from two blots. Normality (quantile–quantile plot) and F-test were first conducted; unpaired *t*-test or Mann–Whitney test was used to compare the groups. ns, not significant. Original Western blot images can be found in Figure S4.

C57BL/6J mice (wild-type, #000664, Jackson Laboratory, Farmington, CT, USA) were purchased and housed in the animal facility at Boston Children’s Hospital (BCH) for at least one week to acclimate to the environment before being used for breeding. In mouse OIR modeling of proliferative ROP, seven-day-old neonatal C57BL/6J mice with their nursing dam were exposed to 75% oxygen [22,23,24] (Figure 1A). Five days later, the mice were returned to room air (21% oxygen) on postnatal day (P) 12. High oxygen exposure leads to vaso-obliteration (Phase I) at the central retina, and relative hypoxia induces physiological re-vascularization (reflected by decreased vaso-obliterated area) and pathological neovascularization (Phase II) [22]. Neovascularization peaks at P17. Retinas were collected for investigation of retinal vaso-obliteration and neovascularization. The vessels were stained with isolectin GS-IB4 (vessel marker, I21413, Invitrogen, Waltham, MA, USA). The images were taken using Zeiss confocal microscopy (Zeiss, White Plains, NY, USA) and were quantified using Image J (1.47v) [23,25,26]. The vaso-obliterated region in the central retina was identified by the lack of red fluorescent signals, whereas the neovascular area was characterized by an accumulation of intense red fluorescence. Both the vaso-obliterated and neovascular regions were quantified and represented as percentages of the total retinal area in the whole mount. The ratio of change was determined by comparing the measured values with the average value of the littermate vehicle control group. All litters were then used for statistical analysis to limit the impact of inter-litter variability of OIR pathology and focused on the drug effects. Both male and female mouse neonates were used. Body weight was recorded.

### 2.3. Folic Acid Deficiency in Maternal Diets

To examine the impact of maternal folic acid deficiency on retinal vasculature, the nursing dams were fed with folic acid deficient (A17082401 (with 1% succinylsulfathiazole to suppress endogenous folic acid synthesis), Research Diets, Inc., New Brunswick, NJ, USA) or control diets with 2 mg/kg folic acid (A07060801, Research Diets, Inc., New Brunswick, NJ, USA) after giving birth. The diet compositions are provided in Table S1. At P12 and P17, retinas were collected for the analysis of vascular status.

### 2.4. Folic Acid Supplementation

Folic acid (F8798, Sigma, St. Louis, MO, USA) was first dissolved in dimethyl sulfoxide (DMSO) to make a 2 µg/µL stock and further diluted to 0.5 µg/µL in phosphate-buffered saline (PBS) on the day of treatment. For intraperitoneal (i.p.) folic acid treatment, the littermate C57BL/6J OIR mouse pups were randomly assigned to either folic acid (0.5 µg/g body weight) or vehicle (25% DMSO in PBS) groups. C57BL/6J OIR mice were treated daily during hyperoxia (P7 to P11, Phase I ROP) or hypoxia (P12 to P16, Phase II ROP). At P12 and P17, retinas were collected for the analysis of vascular status.

### 2.5. Measurement of Total Retinal Folate Levels

Two retinas from the same mouse were pooled for the measurement of total folate concentrations using the microbiological *L. casei* assay as previously described [27,28]. *Lactobacillus casei* growth was quantified at 550 nm by the Epoch Microplate Spectrophotometer (Biotek Instruments, Winooski, VT, USA). Total folate measurements for the retina were normalized to protein concentrations for each sample, as determined using the Lowry–Bensadoun assay [29].

### 2.6. Single-Cell Analysis of Folate Cycle Metabolic Genes

From the single-cell datasets of “Study-Single-cell RNAseq of Normoxic and OIR mouse retina by Drop-seq” (NCBI’s Gene Expression Omnibus accession no. GSE150703) [30], gene expression of enzymes involved in the folate cycle (*Shmt1*, *Shmt2*, *Dhfr*, *Mthfd1*, *Mthfd2*, *Mtr*, *Aldh1l1*, *Aldh1l2*, *Mtfmt*, Figure 1B) in P17 OIR retinal cell types was analyzed using Seurat V4 functions [31] as described in Binet et al., 2020 [30].

### 2.7. Cell Culture

Rat retinal Müller cells (rMC-1) were cultured at 37 degrees Celsius, 5% CO_2_ in a humidified atmosphere in Dulbecco’s Modified Eagle’s Medium (DMEM, #10-0170CV, Corning, New York, NY, USA) supplemented with 10% fetal bovine serum (#S12450, Atlanta Biologicals, Flowery Branch, GA, USA) and 1% antibiotic/antimycotic solution. An equal number of cells per well was plated on 96-well plates (0.5 × 10^4^ cells/well) for MTT cell viability assay (V13154, Invitrogen, Waltham, MA, USA), and 6-well (0.3 × 10^6^ cells/well) plates were used for cell collection for protein and RNA. The culture media was changed, and the cells were treated with vehicle (0.02% DMSO) or 1 µM folic acid for 24 h. Cobalt chloride (CoCl_2_, #15862, Sigma, St. Louis, MO, USA 200 µM) was used to block the degradation of hypoxia-inducible factor 1-alpha (HIF-1α) [32,33,34].

### 2.8. Real-Time PCR

Retinas or rMC-1 cells were lysed with QIAzol lysis reagent and incubated on ice for 15 min. Twenty percent chloroform was added and incubated for 5 min at room temperature. RNA was extracted according to the manufacturer’s instructions using a PureLink^®^ RNA Mini Kit (#12183018A, Ambion, Austin, TX, USA). RNA was then reverse transcribed using the iScript^TM^ cDNA synthesis kit (#1708891, Bio-Rad, Hercules, CA, USA). qPCR was performed for genes involved in the folate cycle, pro- and antioxidants (Table S2). Quantitative analysis of gene expression was generated using an Applied Biosystems 7300 Sequence Detection System with the SYBR Green Master mix kit (Bimak, Huissen, The Netherlands). Gene expression was calculated relative to *Cyclophilin A* using the ΔΔCt method, as we previously reported [35]. The ratio of change was further calculated when compared with the value of control groups. Each sample was repeated in triplicate.

### 2.9. Western Blot

Western blot was conducted as previously reported [36]. Briefly, rMC-1 cells were washed twice with cold PBS and lysed with lysis buffer (RIPA (radioimmunoprecipitation assay, #89900, Pierce, Waltham, MA, USA) + protease inhibitor + phosphatase inhibitor). The protein concentration was determined with bicinchoninic acid (BCA) protein assay kit (#23227, Pierce, Waltham, MA, IL, USA). Equal amounts of proteins (10 µg) from rMC-1 cells were used to detect the levels of HIF-1α (hypoxia-induced factor 1α, 1:500, NB100-479, Novus Biologicals, Centennial, CO, USA) in 5% non-fat milk overnight at 4° Celsius. For retinal lysate, equal amounts of proteins (15 ug) were used to detect MTR (1:1000, 25896, ProteinTech, Rosemont, IL, USA) in 5% non-fat milk overnight at 4° Celsius. Signals were detected using 1:10,000 corresponding horseradish peroxidase-conjugated secondary antibodies and enhanced chemiluminescence (ECL, #34096, Thermo Scientific, Norristown, PA, USA). The digital images were visualized with Azure 600 Imaging System. β-ACTIN (1:2500, #A1978, Sigma, St. Louis, MO, USA) was used as an internal control.

### 2.10. Statistics

Data were presented as mean ± SEM. Normality (quantile–quantile (QQ) plot) and F-test (for variance) were first conducted. A parametric unpaired *t*-test (or Welch’s *t*-test) and a non-parametric Mann–Whitney test was used to compare two groups. ANOVA followed by Tukey’s multiple comparisons test was used to compare multiple groups (Prism v10; GraphPad Software, Inc., San Diego, CA, USA). *p* < 0.05 was considered statistically significant.

## 3. Results

### 3.1. Compromised Expression of Genes Involved in Folate Cycle in OIR Retinas After Hyperoxic Exposure (Phase I)

To test if there were any alterations in the folate cycle (Figure 1B), we first examined the mRNA levels of metabolic genes involved in the folate cycle at P12 (Phase I, hyperoxia) and P17 (Phase II, hypoxia) OIR vs. normal control retinas. Interestingly, decreased gene expression of *Shmt2*, *Mthfd1*, *Aldh1l1*, *Mtfmt*, and *Mtr* was found in P12 OIR vs. control retinas (Figure 1C). No significant change was observed in P17 OIR vs. control retinas (Figure 1D). Protein levels of MTR were also significantly decreased in P12 OIR vs. control retinas (Figure 1E). These findings suggested an affected folate cycle during hyperoxia. We further measured the total folate levels in OIR retinas immediately after hyperoxia at P12; there was no significant change in total folate levels in the retinas (OIR, 36.4 ± 7.1 fmol/µg protein; control, 40.2 ± 7.3 fmol/µg protein; *p* = 0.7117).

### 3.2. Early Folic Acid Deficiency in Maternal Diets Worsened Retinal Neovascularization

As compromised expression of genes involved in the folate cycle was found during hyperoxia (Phase I) and not hypoxia (Phase II), we examined if early folic acid shortage affects pathological retinal angiogenesis. Nursing dams were fed on folic acid-deficient (no folic acid included) or control (2 mg/kg folic acid) diets after giving birth. In OIR, the folic acid-deficient diet was switched to the control diet after hyperoxic exposure (Phase I) at P12. There was more than a 25% drop in total folate levels in P12 OIR mouse retinas from mice fed on folic acid deficient vs. control diets (folic acid deficient, 21.9 ± 1.1 fmol/µg protein, control, 30.6 ± 4.8 fmol/µg protein, *p* = 0.0597).

**Figure 2:**
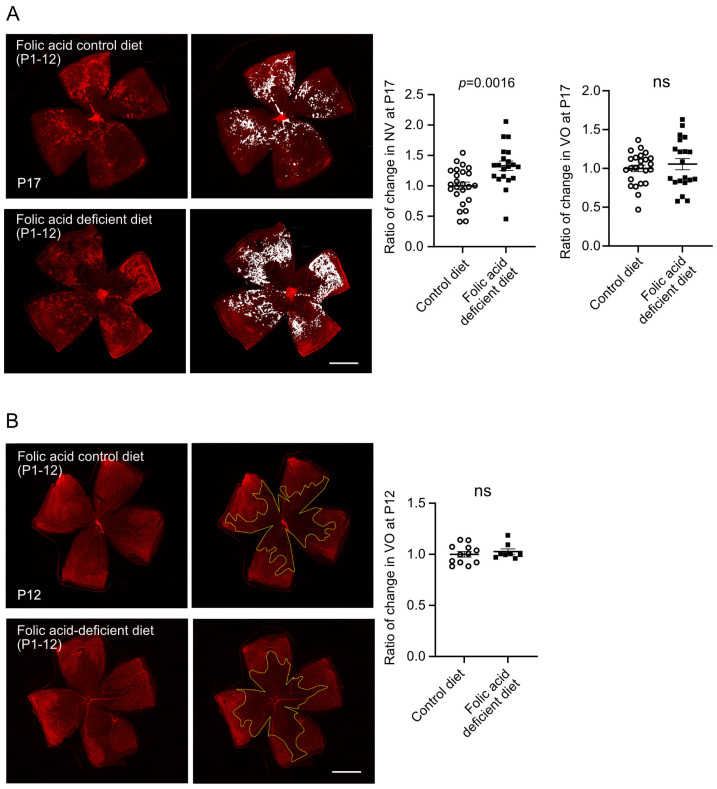
Early folic acid deficiency worsened hypoxia-induced retinal neovascularization in OIR mice. (**A**) Mice were fed on folic acid-deficient or control diet from P1 to P12 and exposed to 75% oxygen from P7 to P12. Mice from both groups were fed on folic acid control diet from P12. At P17, the retinas were collected and stained with isolectin. The neovascular area was highlighted in white. *n* = 20–24 retinas per group from four independent litters (two litters for each diet). (**B**) Mice were fed on folic acid-deficient or control diet from P1 to P12 and exposed to 75% oxygen from P7 to P12. At P12, the retinas were collected immediately after hyperoxia and stained with isolectin. The vaso-obliterated area was outlined in yellow. *n* = 8–12 retinas per group from two independent litters (one litter for each diet). Scale bar, 1 mm. Retinal vaso-obliteration (VO) and neovascularization (NV) were quantified using Image J 1.47v. The ratio of change was calculated and compared with the control group. Normality (quantile–quantile plot) and F-test were first conducted, and unpaired *t*-test (or Welch’s test) or Mann–Whitney test was used to compare the groups. ns, not significant.

For the retinal vascular pathology in OIR, significantly increased retinal neovascularization at P17 was found with an early shortage of maternal dietary folic acid (Figure 2A). Early shortage of folic acid did not affect hyperoxia-induced retinal vaso-obliteration at P12 (Figure 2B). In general, folic acid deficiency did not affect postnatal weight gain (P17, control diet 7.4 ± 0.1 g, folic acid diet 7.2 ± 0.2 g, *p* = 0.4724; P12, control diet 4.6 ± 0.1 g, folic acid diet 4.7 ± 0.1 g, *p* = 0.6454).

### 3.3. Folic Acid Supplementation During Hyperoxia (Phase I) Decreased Retinal Neovascularization

**Figure 3:**
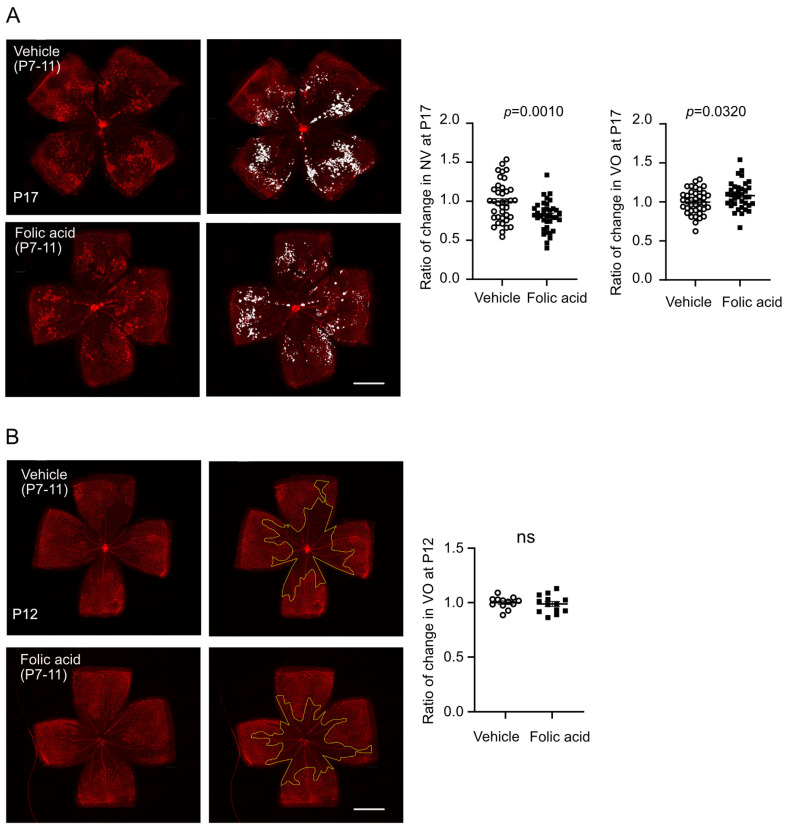
Folic acid supplementation during hyperoxia decreased retinal neovascularization in OIR mice. Mouse pups were treated with either folic acid (0.5 mg/kg, i.p.) or vehicle from P7 to P11. Retinas were collected and stained with isolectin at P17 (**A**) and P12 (**B**). The vaso-obliterated area was outlined in yellow (**B**). Retinal VO and NV were quantified using Image J 1.47v and compared in mice. Scale bar, 1 mm. *n* = 37–38 retinas per group from five independent litters (**A**). *n* = 12–13 retinas per group from two independent litters (**B**). Ratio of change was calculated and compared with littermate control group. Normality (quantile–quantile plot) and F-test were first conducted, and unpaired *t*-test (or Welch’s test) was used to compare the groups. ns, not significant.

**Figure 4:**
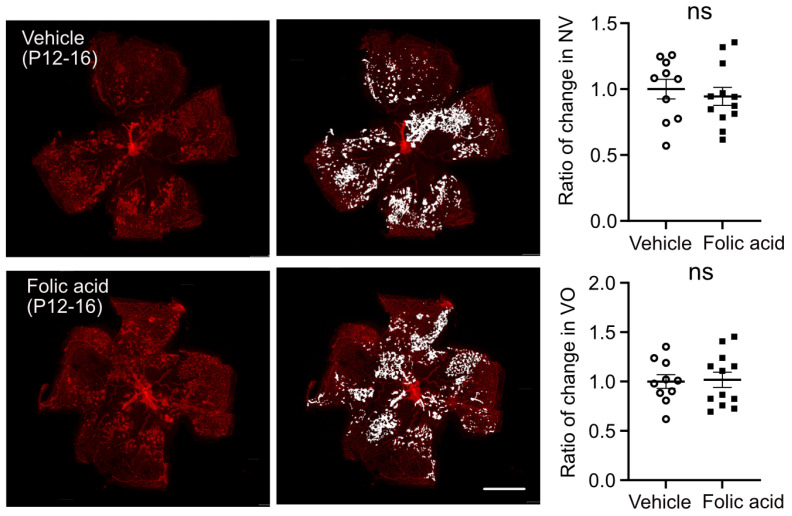
Folic acid supplementation during hypoxia did not change retinal neovascularization in OIR mice. Mouse pups were treated with folic acid (0.5 mg/kg, i.p.) or vehicle from P12 to P16. At P17, the retinas were collected and stained with isolectin. Retinal vaso-obliteration (VO) and neovascularization (NV) were quantified using Image J 1.47v. Scale bar, 1 mm. *n* = 10–12 retinas per group from two independent litters. The ratio of change was calculated and compared with littermate control group. Normality (quantile–quantile plot) and F-test were first conducted, and unpaired *t*-test was used to compare the groups. ns, not significant.

To further examine if supplementation of folic acid during the early stage (hyperoxia, Phase I) protects against retinal neovascularization, littermate OIR mouse pups received either intraperitoneal injection of folic acid or vehicle control daily from P7 to P11 (hyperoxia, Phase I). At P17, there was no significant difference in postnatal weight gain between folic acid- and vehicle-treated groups (*p* = 0.8360). Folic acid treatment (0.5 µg/g) during hyperoxia reduced retinal neovascularization (about 19% decrease in the folic acid-treated group) and slightly delayed retinal re-vascularization (reflected by a larger vaso-obliterated area, about 8% increase in the folic acid-treated group) at P17 (Figure 3A). The body weight was comparable between folic acid and vehicle-treated littermates (vehicle 5.8 ± 0.3 g, folic acid 5.9 ± 0.3 g, *p* = 0.8360). A lower dose of folic acid (0.25 µg/g) during hyperoxia did not reduce retinal pathology in OIR at P17 (VO, *p* = 0.1537, NV, *p* = 0.7739, body weight, *p* = 0.9168). No significant impact of folic acid supplementation (0.5 µg/g) on hyperoxia-induced vaso-obliteration was observed at P12 (Figure 3B), and body weight remained comparable (vehicle 4.6 ± 0.1 g, folic acid 4.5 ± 0.1 g, *p* = 0.5173). Folic acid supplementation during hypoxia (Phase II, P12 to P16, 0.5 µg/g) did not have a significant impact on retinal vasculature (Figure 4) and postnatal weight gain (vehicle 7.0 ± 0.2 g, folic acid 6.8 ± 0.1 g, *p* = 0.9008), in line with our findings with no significant alteration in expression of genes involved in the folate cycle at P17 (hypoxia, Phase II) (Figure 1D).

### 3.4. Folic Acid Decreased Pro-Angiogenic Response in Müller Glia/Astrocytes

**Figure 5:**
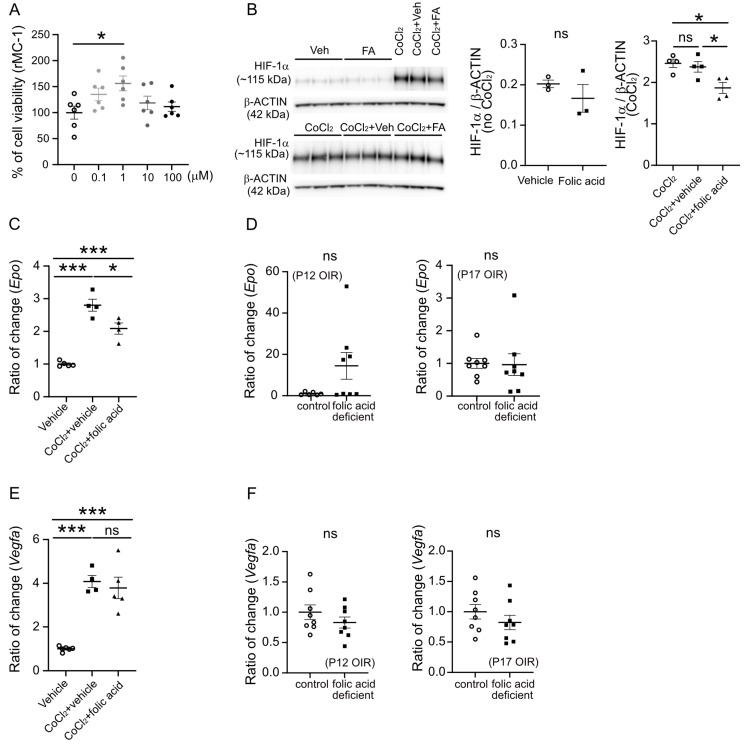
Folic acid decreased Müller glial *Epo* in vitro. Immortalized rat retinal Müller cell line rMC-1 was cultured and treated with folic acid for 24 h. (**A**) Cell proliferation was measured using MTT assay. *n* = 6 per group. ANOVA with Dunnett’s multiple comparison posttest. (**B**) Western blot of HIF-1α in rMC-1 cells treated with folic acid (FA) treatment (1 µM) or vehicle (0.02% DMSO) for 24 h. CoCl_2_ was used to induce HIF-1α stabilization in these cells. *n* = 3–4 per group. The band intensity of HIF-1α was divided by that of β-ACTIN. Unpaired *t*-test or ANOVA. * *p* < 0.05. ns, not significant. Original Western blot images can be found in Figure S5. (**C**) qPCR of rat *Epo* in rMC-1 cells treated with folic acid treatment (1 µM) or vehicle (0.02% DMSO) for 24 h. *n* = 4–5 per group. ANOVA. *** *p* < 0.001, * *p* < 0.05. ns, not significant. (**D**) qPCR of *Epo* in P12 and P17 OIR retinas from mice fed on folic acid-deficient or control diets from P1 to P12. *n* = 8 mice per group. Normality (quantile–quantile plot) and F-test were first conducted, and Mann–Whitney test was used to compare the groups. ns, not significant. (**E**) qPCR of rat *Vegfa* in rMC-1 cells treated with folic acid treatment (1 µM) or vehicle (0.02% DMSO) for 24 h. *n* = 4–6 per group. ANOVA. ns, not significant. (**F**) qPCR of *Vegfa* in P12 and P17 OIR retinas from mice fed on folic acid-deficient or control diets from P1 to P12. *n* = 8 mice per group. Normality (quantile–quantile plot) and F-test were first conducted, and an unpaired *t*-test was used to compare the groups. ns, not significant.

To further examine the retinal cells that may contribute to the folic acid control of neovascularization, we conducted a single-cell analysis of metabolic genes involved in the folate cycle in P17 mouse OIR retinas using a public data source [30]. Amongst all retinal cell types, we found that *Mthfd1*, *Aldh1l1*, and *Aldh1l2* were highly expressed in Müller glia/astrocytes. These cells also expressed other genes involved in the folate cycle, including *Shmt1*, *Shmt2*, *Dhfr*, *Mthfd2*, *Mtr*, and *Mtfmt* (Figure S1). Therefore, we examined the impact of folic acid on rMC-1 cells (immortalized rat Müller cells) in vitro. Folic acid treatment (1µM) increased the cell viability of rMC-1 (Figure 5A).

We further examined whether HIF-1α, a transcriptional factor that controls pro-angiogenic erythropoietin (*Epo*) expression [37], was a target of folic acid. Folic acid has been known to reduce HIF activities in ocular cells (ARPE-19) [38]. HIF-1α protein was stabilized under the hypoxic condition and instantly increased within 2 h in neonatal mouse retinas after returning to room air in the OIR model [39]. Inhibition of *Epo* mRNA expression suppressed retinal neovascularization in OIR [40]. Folic acid treatment did not affect HIF-1α levels in rMC-1 cells under normoxic or naïve conditions. The treatment of CoCl_2_ induced HIF-1α stabilization in rMC-1 cells, and folic acid treatment reduced its induction (Figure 5B). The increased expression of *Epo*, under CoCl_2_-incubating conditions or by CoCl_2_-triggered pseudohypoxic stress, was also decreased by folic acid treatment (Figure 5C). However, there was a trend toward increased total retinal expression of *Epo* in the folic acid-deficient vs. control diet-fed groups at P12, and no significant change was observed at P17 (Figure 5D). This suggested that total retinal responses might not reflect potential cell-specific responses to folic acid modulation. We did not find significant impacts of folic acid on the other HIF-1α downstream factor *Vegfa* expression in rMC-1 cells (Figure 5E) and OIR retinas (Figure 5F).

As the folate cycle plays a key role in controlling redox homeostasis [41,42], we also quantified mRNA levels of antioxidants (*Cat*, *Prdx1*, *Sod2*, *Trx2*, *Gpx1*, *Grx1*) and prooxidants (*Cox2*, *iNos*) in P12 and P17 neonatal mouse OIR retinas from the nursing dams fed on folic acid-deficient vs. control diets. No significant differences were found between the two groups (Figure S2A,B). Moreover, folic acid-deficiency in the maternal diet did not exert significant impacts on folate cycle genes except for *Aldh1l1*, which was decreased with folic acid deficiency (Figure S3).

## 4. Discussion

Early deficiency of folic acid could occur in premature infants, but the consequences on retinal development remain unknown. We demonstrated that decreased maternal dietary intake of folic acid after delivery and during Phase I hyperoxia exacerbated pathological retinal angiogenesis in OIR. Direct supplementation of folic acid to mouse neonates during Phase I hyperoxia (P7 to P11) but not Phase II hypoxia (P12 to P16) suppressed neovascularization. These findings corresponded to compromised gene expression of proteins involved in the folate cycle at Phase I (P12, immediately after hyperoxia), and the decrease was not found at Phase II (P17) OIR retinas.

Increasing circulating folate levels in premature infants is very safe and feasible by either direct supplementation to the babies or by increasing maternal intake of folic acid. Low-birth-weight infants (~1800 g at ~33 weeks of gestation) receiving 25 µg folic acid immediately after birth up to one month of age (or when the baby reaches 2500 g) have higher serum folate levels than the basal levels [9]. Oral supplementation of folic acid (100 µg, daily) given after the first month of age shows a marked rise in serum folate levels in premature infants [8]. The breast milk folate levels correlate with maternal intake of folic acid [9]. Premature infants (≤32 weeks of gestation) fed on human breast milk (150 ng/mL folic acid) alone have lower serum folate levels than those fed on fortified human breast milk (500 ng/mL) or preterm formula (350 ng/mL folic acid) [12]. Impaired folate pathways cause many eye symptoms [15,16,17,18]. Our current study highlighted that folic acid deficiency in early life worsened retinal neovascularization in a mouse model of ROP. Therefore, increasing folic acid supplementation is necessary to increase the folate levels in infants to prevent abnormal retinal development. However, the dose of folic acid needs to be tightly controlled. Here, we showed that folic acid supplementation not only remarkably suppressed pathological retinal vessel growth but also caused a slight delay in physiological retinal angiogenesis.

Müller glial cells (~3.6% of cells in mouse retina) span the retina [43]. With hypoxia or fuel shortage, Müller glia cells release neurotrophic/angiogenic factors, such as VEGF, which help maintain retinal function and promote vascular growth [44]. VEGF is highly induced in Müller glia after vessel loss in OIR [22]. Loss of VEGF in Müller glia inhibits retinal neovascularization in OIR [45,46]. We found folic acid supplementation did not change Müller glia *Vegfa* in vitro and *Vegfa* expression in OIR retinas in vivo, suggesting the inhibitory effects of folic acid on retinal neovascularization were likely independent of VEGF signaling. Retinal *Epo* expression was also regulated by HIF-1α [37]. *Epo* mRNA expression was induced during the hypoxic phase in mouse OIR retinas [40]. Intravitreal injection of *Epo* siRNA at P12 significantly decreased *Epo* expression at P14 and retinal neovascularization at P17 in mouse OIR [40]. We observed a decreased Müller glial expression of *Epo* in vitro with folic acid supplementation and a trend of increased retinal *Epo* expression with folic acid shortage at P12 in vivo, suggesting that folic acid might downregulate HIF-1α/EPO signaling to decrease retinal neovascularization.

Since it was early supplementation of folic acid (hyperoxic phase), not late supplementation (hypoxic phase), that suppressed retinal neovascularization and HIF-1α signaling induced under hypoxia, we speculated that folic acid might be key in modulating hypoxic responses induced immediately after hyperoxia. HIF-1α is strongly increased within 2 h after hypoxic exposure in mouse OIR retinas and decreased within 24 h [39]. Therefore, the appropriate amount of retinal folate on the first day of hypoxia might play an essential role in modulating hypoxia-induced HIF-1α stabilization and, in turn, *Epo* expression. However, it may take several days to induce accumulated change in the retinal level of folate. We have found that there was only a 25% drop (*p* = 0.0597) in retinal folate levels in OIR mice with a dietary maternal folic acid deficiency for 12 postnatal days.

Premature infants are highly susceptible to reactive oxidative species-induced damage as increased synthesis of the antioxidants occurs during the last 15% of gestation [47,48], which is disrupted in these infants due to premature birth. Redox imbalance is associated with increased ROP risk and severity [49,50,51]. In rodents modeling ROP, the administration of antioxidants such as nicotinamide adenine dinucleotide phosphate oxidase inhibitor [52], vitamin E [53], and genetically increased superoxide dismutase activity [54] inhibits retinal vascular pathology. One carbon metabolism, mediated by the folate cofactors, controls cell redox homeostasis [41,42,52]. Folic acid supplementation effectively antagonizes hyperhomocysteinemia-induced oxidative stress, restores superoxide dismutase activity in rats, and reduces superoxide anion production in vitro [53]. However, there were no significant changes in gene expression of antioxidants and prooxidants in total retinas from folic acid-deficient vs. control diet-fed groups. A better understanding of the cell-specific impacts of folic acid on retinal development could further expand our knowledge of the underlying mechanism.

There were some limitations in our current study. First, due to the limitation of the mouse OIR model, in which retinal pathology is induced in full-term mice, OIR mice have the limitation of fully reflecting the nutrient deprivation and the pathogenesis in preterm babies developing ROP who are at a high risk of folate deficiency. Moreover, our current method using the microbiological *L. casei* assay to measure total folate levels in the retina has limitations in looking at different forms of folate. It was still unclear if there was a deficiency in folate levels during hyperoxia. The deficiency in folate metabolism reflected by decreased folate cycle genes and proteins suggested hyperoxia may affect the efficiency of folate use in OIR retinas. However, inhibition of DHFR using methotrexate (0.2 μg/g, i.p. P6–P11) during hyperoxia did not affect vaso-obliteration and neovascularization in mouse OIR (PMID: 31341109). It was unclear if a higher dose of methotrexate delivered during hyperoxia would lead to worsened OIR pathology. Last but not least, rMC-1 expresses glial fibrillary acidic protein (GFAP, a marker for Müller glial activation) in the culturing system, and hypoxia caused about a 1.3-fold decrease in *Gfap* gene expression [54]. However, GFAP in OIR Müller glia is induced during hypoxia [22]. rMC-1 does not have contact with other retinal cells and only partially mimics the in vivo condition. Folic acid may directly impact HIF-1α levels in hypoxia-induced endothelial cells [55]. Further investigation of folic acid on specific retinal cell responses in in vivo conditions would greatly expand our understanding of the mechanisms behind folic acid suppression on retinal neovascularization and accelerate the clinical practice of folic acid supplementation to prevent ROP.

## 5. Conclusions

Our work highlights the importance of folic acid in early life for controlling pathological retinal angiogenesis and suggests that timely supplementation of folic acid may help prevent ROP.

## Data Availability

The datasets generated during and/or analyzed during the current study are available from the corresponding author on reasonable request.

## Supplementary Materials

The following supporting information can be downloaded at https://www.mdpi.com/article/10.3390/biom15020309/s1, Figure S1: Metabolic genes involved in the folate cycle were expressed in Müller glia/astrocytes; Figure S2: Gene expression of anti- and prooxidants in OIR mice fed with control or folic acid deficient diet; Figure S3: Gene expression of folate cycle genes at OIR mice fed with control or folic acid deficient diet; Figure S4: Original Western blot images of Figure 1E blot 1 (A) and 2 (B); Figure S5: Original Western blot images of Figure 5B blot 1 (A) and 2 (B); Table S1: Compositions of folic acid control and deficient diets; Table S2: Primers of genes for real-time PCR.

## Abbreviations

ALDH1L1	10-formyltetrahydrofolate dehydrogenase family member L1
ALDH1L2	10-formyltetrahydrofolate dehydrogenase family member L2
BCA	bicinchoninic acid
Cat	catalase
CoCl_2_	cobalt chloride
Cox2	cyclooxygenase-2
CycloA	cyclophilin a
DHFR	dihydrofolate reductase
DMEM	Dulbecco’s modified Eagle’s medium
DMSO	dimethyl sulfoxide
ECL	enhanced chemiluminescence
FA	folic acid
Epo	erythropoietin
GFAP	glial fibrillary acidic protein
Gpx1	glutathione Peroxidase 1
Grx1	glutaredoxin-1
HIF1α	hypoxia-induced factor 1α
iNOS	nitric oxide synthase, inducible
MTFMT	mitochondrial methionyl-tRNA formyltransferase
MTHFD1	methenyltetrahydrofolate cyclohydrolase 1
MTHFD2	methenyltetrahydrofolate cyclohydrolase 2
MTHFR	methylenetetrahydrofolate reductase
MTR	methionine synthase
NV	neovascularization
OIR	oxygen-induced retinopathy
PBS	phosphate-buffered saline
Prdx1	peroxiredoxin 1
RIPA	radioimmuoprecipitation assay
rMC-1	rat Müller cell-1
ROP	retinopathy of prematurity
SHMT1	serine hydroxymethyltransferase 1
SHMT2	serine hydroxymethyltransferase 2
Sod2	superoxide dismutase 2
Trx2	thioredoxin 2
VEGF	vascular endothelial growth factor
VO	vaso-obliteration
