# Supplementary material for: Folic Acid Supplementation Inhibits Proliferative Retinopathy of Prematurity
Source: Biomolecules. 2025 Feb 19;15(2):309. doi: 10.3390/biom15020309 (PMC11852370; doi:10.3390/biom15020309)
Supplement: Supplementary file 1 [file biomolecules-15-00309-s001.zip › biomolecules-3384049-supplementary.pdf]

Supplementary Materials

Table S1. Compositions of folic acid control and deficient diets. Succinylsulfathiazole was added to minimize the formation of folic acid by bacteria. Sucrose was used as "filler" ingredients used when replacing folic acid in the diet, which was 2 mg per kg diet.

Product #	D10012G	A07060801B	A17082401R
	AIN-93G	L-Amino Acid	No Added Folate and
		AIN-93G	w/ Succinylsulfa.
	gm%	gm%	gm%
**Ingredient**	gm	gm	gm
L-Arginine	0	6	6
L-Histidine-HCl-H2O	0	4.6	4.6
L-Isoleucine	0	7.6	7.6
L-Leucine	0	15.8	15.8
L-Lysine-HCl	0	13.2	13.2
L-Methionine	0	5.1	5.1
L-Phenylalanine	0	8.4	8.4
L-Threonine	0	7.2	7.2
L-Tryptophan	0	2.1	2.1
L-Valine	0	9.3	9.3
L-Alanine	0	5.1	5.1
L-Asparagine-H2O	0	6.7	6.7
L-Aspartate	0	5.4	5.4
L-Cystine	3	4.2	4.2
L-Glutamic Acid	0	21.7	21.7
L-Glutamine	0	16.5	16.5
Glycine	0	3	3
L-Proline	0	17.8	17.8
L-Serine	0	10	10
L-Tyrosine	0	9.2	9.2
Total L-Amino Acids	3	178.9	178.9
Casein	200	0	0
Corn Starch	397.486	397.486	397.486
Maltodextrin 10	132	132	132
Sucrose	107.0777	107.0777	107.0777
Cellulose	50	50	50
Soybean Oil	70	70	70
t-butylhydroquinone	0.014	0.014	0.014
Mineral Mix S10022G	0	0	0
Mineral Mix S10022C	3.5	3.5	3.5
Calcium Carbonate	12.495	7.34	7.34
Potassium Citrate, 1 H20	2.4773	2.4773	2.4773
Potassium Phosphate, Monobasic	6.86	6.86	6.86
Calcium Phosphate, dibasic	0	7	7
Sodium Chloride	2.59	2.59	2.59
Sodium Bicarbonate	0	7.5	7.5
Vitamin Mix V10037 (w/ Folic Acid)	10	10	0
Vitamin Mix V14905 (w/o Folic Acid)	0	0	10
Choline Bitrartrate	2.5	2.5	2.5
Succinylsulfathiazole	0	0	10
FD&C Yellow Dye #5	0	0	0
FD&C Blue Dye #1	0	0.05	0
FD&C Red Dye #40	0	0	0.05
**Total**	**1000**	**985.245**	**995.295**

Table S2. Primers of genes for real-time PCR.

Gene	Forward Primer	Reverse Primer
Mouse		
Dhfr	CGCTCAGGAACGAGTTCAAGT	TGCCAATTCCGGTTGTTCAATA
Shmt1	TATTATGGCGGGACCGAGTTC	GAGGCTCTACCAGGGCAGTA
Shmt2	TGGCAAGAGATACTACGGAGG	AGATCCGCTTGACATCAGACA
Mthfd1	GGGAATCCTGAACGGGAAACT	TGAGTGGCTTTGATCCCAATC
Mthfd2	AGTGCGAAATGAAGCCGTTG	GACTGGCGGGATTGTCACC
Mtr	ATGATCCAGCGGTACAAACTAAG	CATCCGGTAGGCCAAGTGTTC
Aldh1l1	AGCCACCTATGAGGGCATTC	TGAGTGTCGAGTTGAAAAACGTC
Aldh1l2	ACCAGCCGGGTTTATTTCAAA	ACTCCCACTACTCGGTGGC
Mtfmt	TTCCCTACGGCATACTGAATGT	CACCGTGTGGATTATCGGAGC
Cat	AGCGACCAGATGAAGCAGTG	TCCGCTCTCTGTCAAAGTGTG
Epo	AGGAATTGATGTCGCCTCCA	AGCTTGCAGAAAGTATCCACTGTG
Gpx1	GGGACTACACCGAGATGAACGA	ACCATTCACTTCGCACTTCTCA
Trx2	GGACCGCGGCTAGAGAAGAT	GCTGGTCCTCGTCCTTGATC
Grx1	CAACACCAGTGCGATTCAAGA	GCAGAGCTCCAATCTGCTTCA
Prdx1	ACACCCAAGAAACAAGGAGGATT	CAACGGGAAGATCGTTTATTGTTA
iNOS	GGAGTGACGGCAAACATGACT	TCGATGCACAACTGGGTGAAC
Sod2	CAGACCTGCCTTACGACTATGG	CTCGGTGGCGTTGAGATTGTT
Cox2	TGAGCAACTATTCCAAACCAGC	GCACGTAGTCTTCGATCACTATC
Vegfa	GGAGACTCTTCGAGGAGCACTT	GGCGATTTAGCAGCAGATATAAGAA
CycloA	CAGACGCCACTGTCGCTTT	TGTCTTTGGAACTTTGTCTGCAA
Rat		
Epo	CGAACGTCCCACCCTGCTGC	GTGGGGGAGCGCAGAGGACT
Vegfa	GCTCCTTCACTCCCTCAAATTA	GGTCTCTCTCTCTCTCTCTCTTC
CycloA	CTTTGCAGACGCCGCTGTCTCTT	CTGCTGTCTTTGGAACTTTGTCTGC

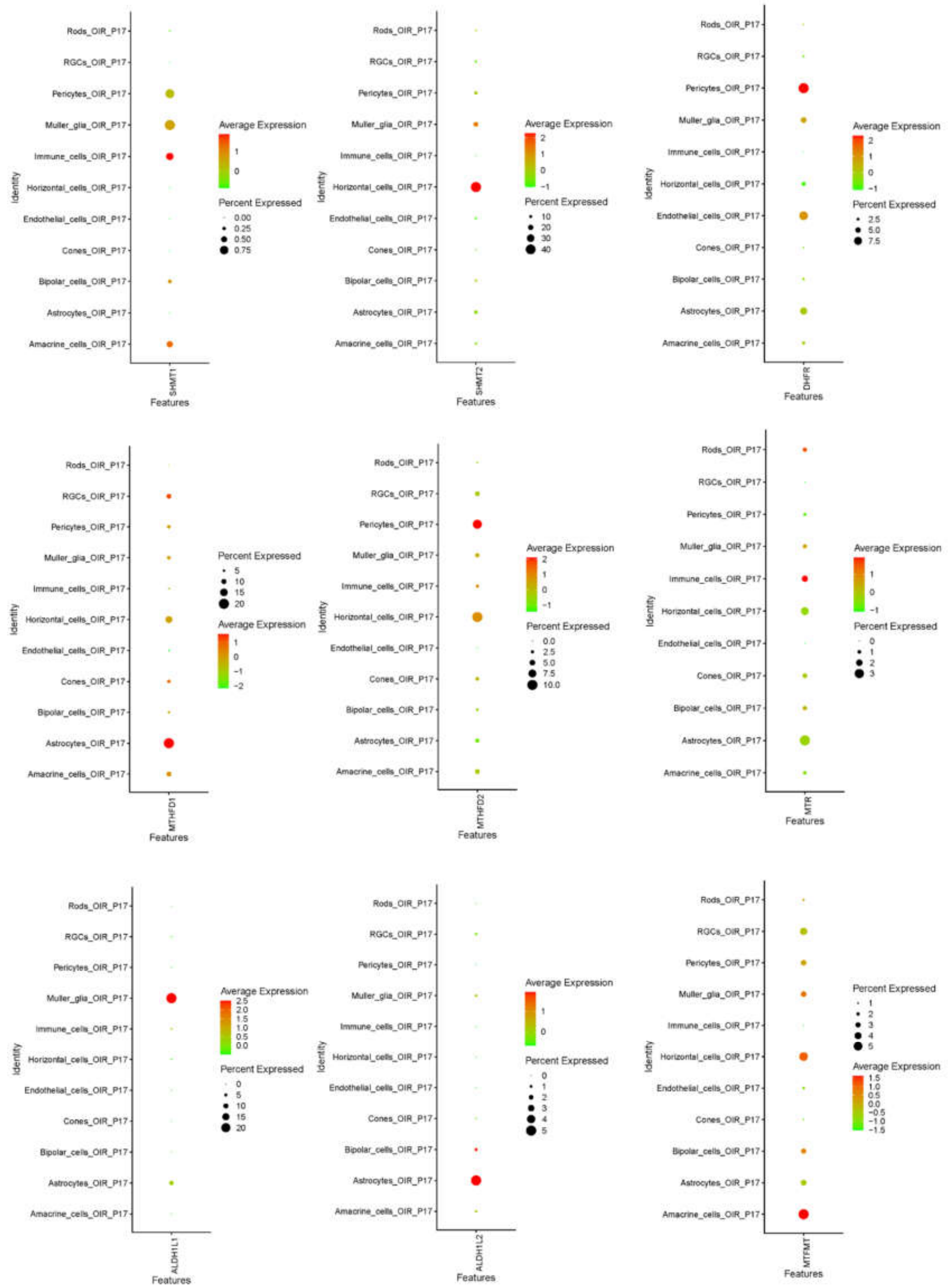

Figure S1. Metabolic genes involved in folate cycle were expressed in Müller glia/astrocytes. Gene expression of folate cycle metabolic enzymes with single-cell transcriptomic analysis of P17 OIR mouse retinas. RPE was removed from the retina before single cell isolation. Per. exp is the percent of cells that have one or more transcript for the gene of interest; avg exp. is the average transcript count amongst the cells of a cluster. Transcript counts are scaled around zero (zero = the mean across all cells of the dataset).

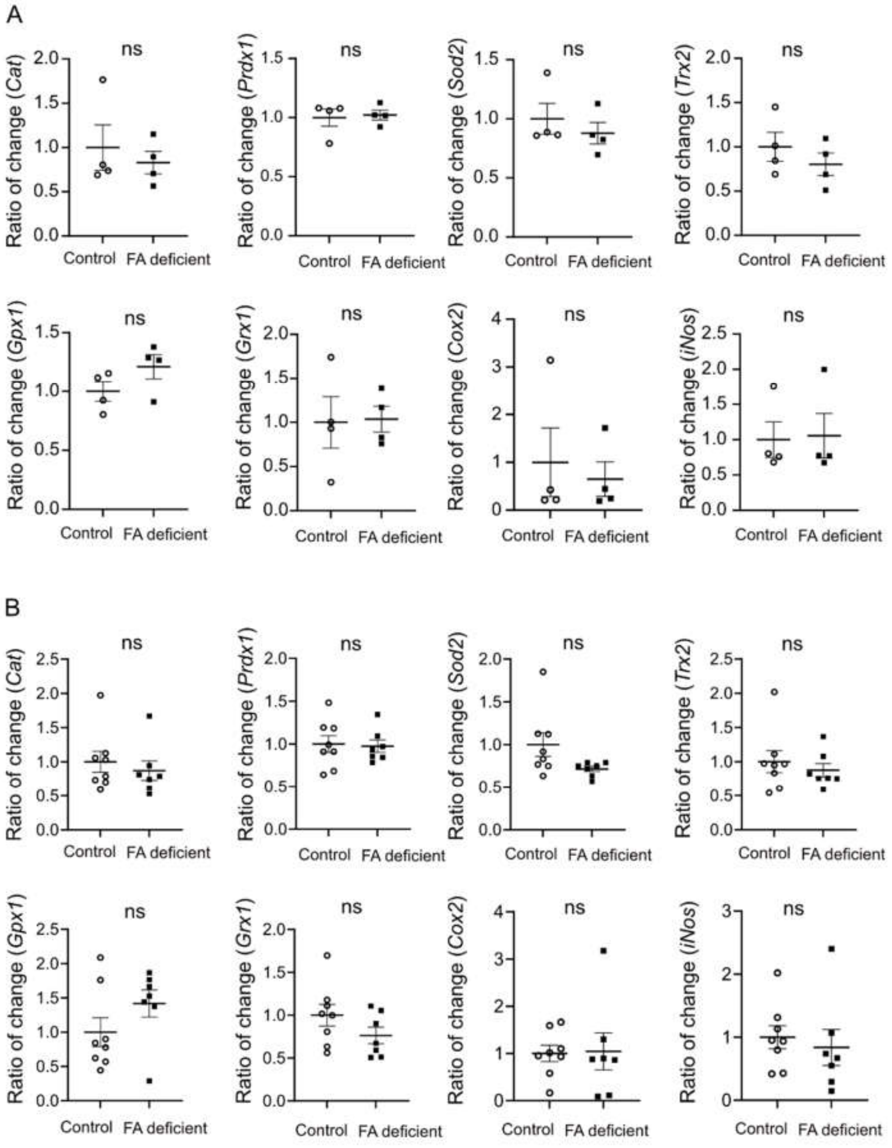

Figure S2. Gene expression of anti- and pro-oxidants at OIR mice fed with control or folic acid deficient diet. Mice were fed on folic acid deficient or control diet from P1 to P12 and exposed to 75% oxygen from P7 to P12. Mice from both groups were fed on folic acid control diet from P12. Retinas at P12 (**A**) and P17 (**B**) were used for examining antioxidants (*Cat, Prdx1, Sod2, Trx2, Gpx1, Grx1*) and prooxidants (*Cox2, iNos*) with qPCR. n=4 mice per group (**A**), n=7-8 mice per group (**B**). Normality (quantile-quantile plot) and F-test was first conducted, unpaired t-test or Mann-Whitney test was used to compare the groups. ns, not significant.

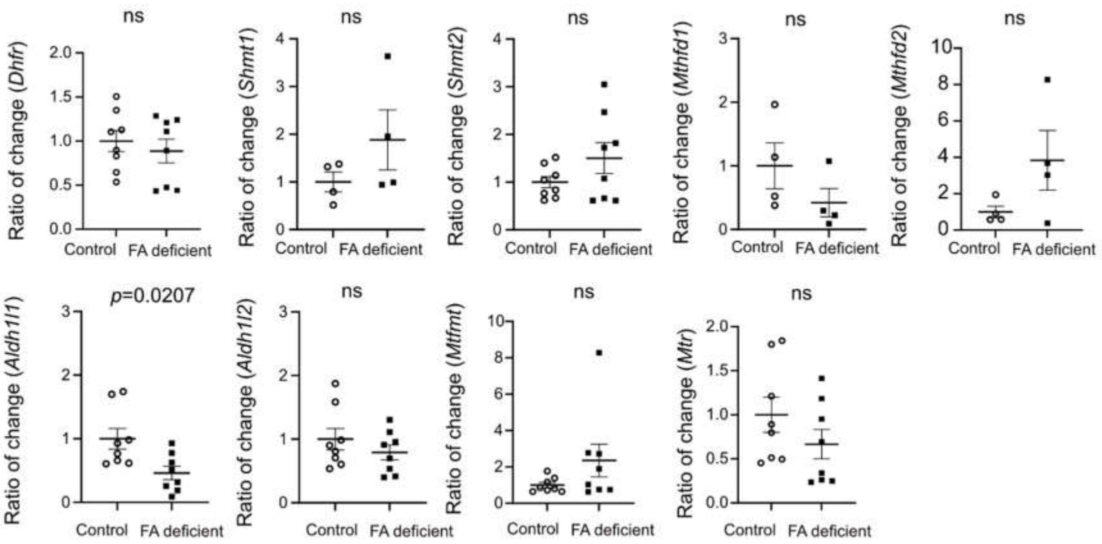

Figure S3. Gene expression of folate cycle genes at OIR mice fed with control or folic acid deficient diet. Mice were fed on folic acid deficient or control diet from P1 to P12 and exposed to 75% oxygen from P7 to P12. Mice from both groups were fed on folic acid control diet from P12. n=4 mice per group (*Shmt1, Mthfd1, Mthfd2*), n=8 mice per group for other genes. Normality (quantile-quantile plot) and F-test was first conducted, unpaired t-test or Mann-Whitney test was used to compare the groups. ns, not significant.

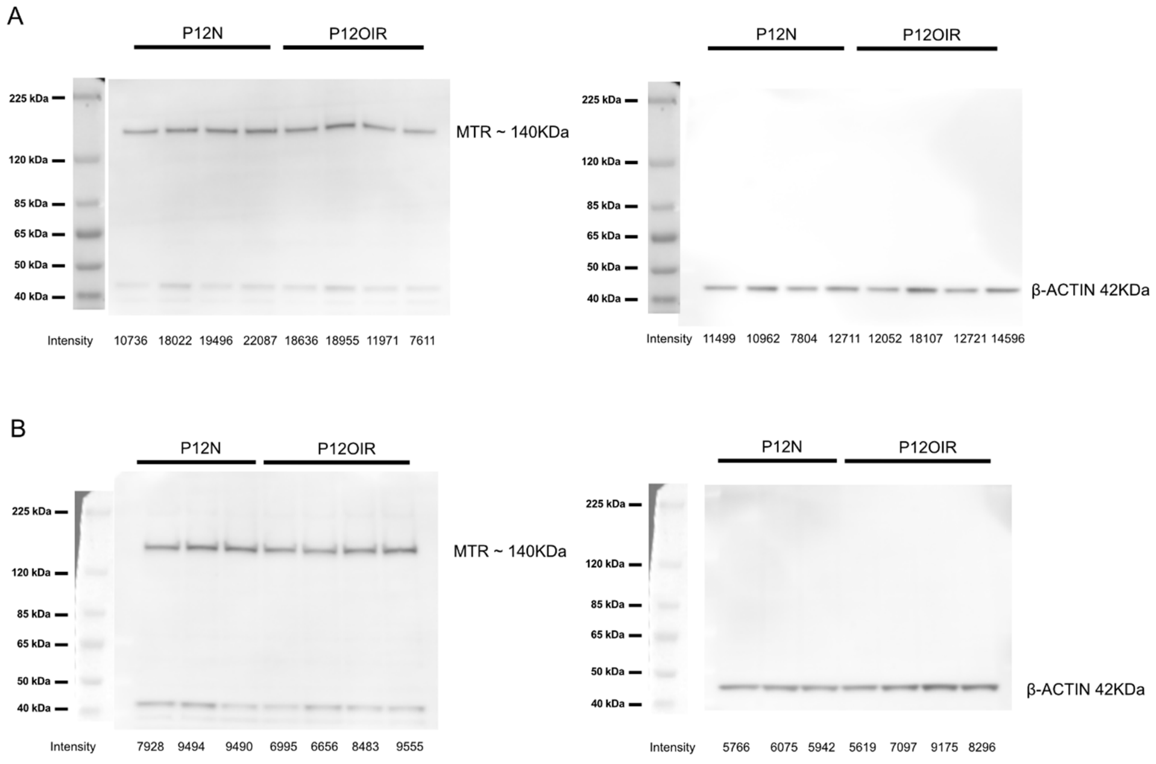

Figure S4. Original Western blot images of Figure 1E blot 1 (**A**) and 2 (**B**).

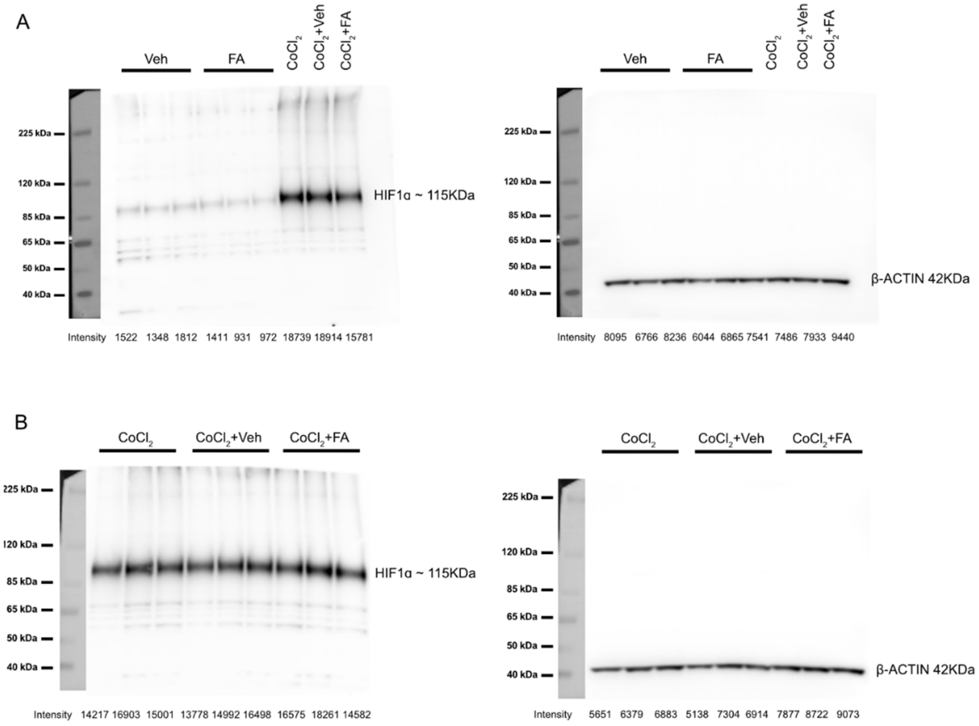

Figure S5. Original Western blot images of Figure 5B blot 1 (**A**) and 2 (**B**).
